# Plasma and CSF neurofilament light: Relation to longitudinal neuroimaging and cognitive measures

**DOI:** 10.1212/WNL.0000000000007767

**Published:** 2019-07-16

**Authors:** Michelle M. Mielke, Jeremy A. Syrjanen, Kaj Blennow, Henrik Zetterberg, Prashanthi Vemuri, Ingmar Skoog, Mary M. Machulda, Walter K. Kremers, David S. Knopman, Clifford Jack, Ronald C. Petersen, Silke Kern

**Affiliations:** From the Departments of Health Sciences Research (M.M. Mielke, J.A.S., W.K.K., R.C.P., S.K.), Neurology (M.M. Mielke, D.S.K., R.C.P.), Radiology (P.V., C.J.), and Psychiatry and Psychology (M.M. Machulda), Mayo Clinic, Rochester, MN; Department of Psychiatry and Neurochemistry (K.B., H.Z., I.S., S.K.), Institute of Neuroscience and Physiology, The Sahlgrenska Academy at the University of Gothenburg; Clinical Neurochemistry Laboratory (K.B., H.Z.), Sahlgrenska University Hospital, Mölndal, Sweden; Institute of Neurology (H.Z.), University College London, Queen Square; and UK Dementia Research Institute at UCL (H.Z.), London, UK.

## Abstract

**Objective:**

We aimed to (1) assess and compare baseline plasma and CSF neurofilament light (NfL) for cross-sectional and longitudinal associations with neuroimaging or cognition and (2) determine whether change in plasma NfL corresponded with change in these outcomes.

**Methods:**

Seventy-nine participants without dementia, median age 76 years, had plasma and CSF NfL, neuropsychological testing, and neuroimaging (MRI, amyloid PET, FDG-PET) at the same study visit, and a repeat visit (15 or 30 months later) with both plasma NfL and neuroimaging. Plasma NfL was measured on the Simoa-HD1 Platform and CSF NfL with an in-house ELISA. Linear mixed effects models were used to examine the associations between baseline plasma or CSF NfL and cognitive and neuroimaging outcomes adjusting for age, sex, and education. The relationship between change in plasma NfL and change in the outcomes was assessed using linear regression.

**Results:**

There were no cross-sectional associations between CSF or plasma NfL and any neuroimaging or cognitive measure. Longitudinally, higher baseline plasma NfL was associated with worsening in all neuroimaging measures, except amyloid PET, and global cognition. Higher baseline CSF NfL was associated with worsening in cortical thickness and diffusion MRI. The beta estimates for CSF NfL were similar to those for plasma NfL. Change in plasma NfL was associated with change in global cognition, attention, and amyloid PET.

**Conclusion:**

Elevated baseline plasma NfL is a prognostic marker of cognitive decline and neuroimaging measures of neurodegeneration, and has similar effect sizes to baseline CSF NfL. Change in plasma NfL also tracked with short-term cognitive change.

Blood-based biomarkers of neurodegeneration have obvious advantages over both CSF and neuroimaging biomarkers. The collection of blood is inexpensive, noninvasive, and a more feasible measure for use in the general population, especially if serial collection is needed. Neurofilament light (NfL) is a recognized biomarker of subcortical large-caliber axonal degeneration.^1,2^ CSF and plasma NfL levels are elevated in multiple neurodegenerative disorders, including Alzheimer disease (AD) dementia,^3,4^ frontotemporal dementia,^5^ multiple sclerosis,^6^ and traumatic brain injury.^7,8^ Although the NfL elevation is lower in AD dementia compared to other neurodegenerative disorders (e.g., vascular dementia and frontotemporal dementia),^9,10^ NfL is hypothesized to be a nonspecific marker of neurodegeneration. Indeed, among participants who are either cognitively unimpaired (CU) or have mild cognitive impairment (MCI), the longitudinal relationship between plasma or CSF NfL and cognitive or imaging measures of neurodegeneration are independent of elevated brain amyloid.^3,4^

To date, longitudinal studies of NfL across the AD clinical spectrum have focused on either CSF or plasma and have not compared the effect sizes for change in neuroimaging or cognitive measures. Thus, it is not known how similarly plasma NfL reflects the prognostic value of CSF NfL for cognitive decline and neurodegeneration. In addition, studies have primarily examined plasma NfL at one point in time and have not determined whether change in plasma NfL associates with change in cognitive or neuroimaging measures. The objectives of this study were to (1) assess and compare the cross-sectional and longitudinal associations between concurrent baseline measures of plasma or CSF NfL with neuroimaging (amyloid PET [^18^F]-fluorodeoxyglucose [FDG]–PET, MRI) or global- and domain-specific cognitive *z* scores; and (2) determine whether change in plasma NfL corresponded with change in these outcomes.

## Methods

The Mayo Clinic Study of Aging (MCSA) is a population-based, prospective study of residents living in Olmsted County, Minnesota.^11^ In 2004, the Rochester Epidemiology Project (REP) medical records linkage system was used to enumerate Olmsted County residents between the ages of 70 and 89, as previously described.^12^ In 2012, the MCSA was extended to include those aged 50 and older. The present analyses included all 79 participants without dementia (64 CU, 15 MCI) with concurrent measures of both plasma and CSF NfL, neuropsychological testing, and neuroimaging (amyloid Pittsburgh compound B [PiB]–PET, FDG-PET, MRI) at the same study visit. All participants were also required to have a repeat clinical visit that included both the measurement of plasma NfL and neuroimaging.

### Standard protocol approvals, registrations, and patient consents

The institutional review boards of both Mayo Clinic and Olmsted Medical Center approved this study. All participants provided written informed consent.

### Participant assessment

MCSA clinical visits occurred every 15 months.^11^ These visits included an interview by a study coordinator/nurse, a neurologic examination, and neuropsychological testing.^11^ The neuropsychological battery included 9 tests covering 4 domains (memory, language, executive function, and visuospatial), as previously described.^11^ Among the CU, sample-specific *z* scores were computed for each cognitive test. Domain scores were then estimated by averaging the *z* scores within each domain. A global cognitive *z* score was calculated by averaging the 4 domain *z* scores.

### MCI and dementia diagnostic determination

Clinical diagnoses were determined by a consensus committee including the study coordinator, neuropsychologist, and the physician who evaluated each participant, as previously described.^11^ Performance in a cognitive domain was compared with the age-adjusted scores of CU participants previously obtained using Mayo's Older American Normative Studies in an independent sample from the same county.^13^ Participants were considered for possible cognitive impairment if their scores were around 1.0 SD below the age-specific mean in the general population. The operational definition of MCI was based on clinical judgment, including a history from the patient and informant, using published criteria.^14^ A final decision about impairment in a cognitive domain was made after considering education, occupation, visual or hearing deficits, and all other participant information. The diagnosis of dementia was based on published criteria.^15^ Participants who performed in the normal range and did not meet criteria for MCI or dementia were deemed CU. Neither CSF results nor neuroimaging were considered in determining the clinical diagnoses of MCI or dementia.

### CSF NfL

Fasting lumbar punctures were performed early in the morning in the lateral decubitus position using a 20- or 22-gauge Quincke needle, as previously described.^4^ Two milliliters of CSF were used to evaluate routine markers (glucose, protein, cell count). The remainder was divided into 0.5-mL aliquots and stored at −80°C for future analyses avoiding freeze–thaw cycles prior to the current analyses.^4^ CSF NfL was measured in the Clinical Neurochemistry Laboratory at the University of Gothenburg, Mölndal, Sweden, using an in-house sandwich ELISA with capture and detection antibodies directed against the central rod domain of the protein (NfL21 and NfL23, respectively).^16^ As published, this ELISA has within-plate and interplate variations of below 8% and 13%, respectively, and a strong correlation (*r* = 0.9984, *p* < 0.001) with CSF values analyzed using Uman Diagnostics ELISA.^16^

### Plasma NfL

Blood was collected in EDTA tubes in clinic after an overnight fast.^4^ As previously described, the blood was centrifuged, aliquoted in 500 μL aliquots, and stored at −80°C for future analyses, thus avoiding freeze–thaw cycles prior to the current analyses.^4^ Plasma NfL was measured using an in-house digital ELISA on the Simoa-HD1 Platform, as previously described.^17^ Intra-assay and interassay coefficients of variation were 11.7%–12.1% for quality control samples with clinically relevant low and high concentrations (17.9 and 257 pg/mL, respectively). The analytical sensitivity was 0.62 pg/mL.^4^ The validated measurement range was 6.7–1,620 pg/mL.^4^

### Neuroimaging variables

Neuroimaging occurs at 15- or 30-month intervals. The acquisition, processing, and summary measure for AD signatures for amyloid PET, FDG-PET, and MRI in the MCSA have been described in detail.^18–20^ Briefly, a global cortical PiB-PET retention ratio was computed by calculating the median uptake over voxels in the anterior cingulate, orbitofrontal, parietal, prefrontal, posterior cingulate/precuneus, and temporal regions. This summary value was then divided by the median uptake over voxels in the cerebellar gray matter atlas region.^21^ We classified participants as having elevated brain amyloid (A+) if their PiB-PET standardized uptake value ratio (SUVR) was ≥1.42. A global FDG-PET ratio measure was computed for each individual scan by averaging the left and right angular gyri, bilateral posterior cingulate, and left middle/interior temporal gyrus pons-normalized SUVR values for each participant, as described previously.^20^ Using our in-house fully automated imaging processing pipeline, hippocampal volume was adjusted for total intracranial volume.^22^ A global cortical thickness AD measure was computed using a FreeSurfer (version 5.3)–derived temporal lobe cortical thickness composite of entorhinal, fusiform, inferior temporal, and middle temporal regions of interest^20^ from 3T magnetization-prepared rapid gradient echo scans. Diffusion tensor imaging (DTI) sequences were processed and analyzed for fractional anisotropy (FA) of the corpus callosum.^23^ Loss of white matter microstructural integrity measured using DTI is a good indicator of axonal injury. We used FA in the corpus callosum as an imaging marker of axonal damage, which has previously been shown to be a sensitive measure of axonal injury.^24,25^

### Assessment of covariates

Age, sex, and years of education were obtained at the clinical visit. *APOE* ε4 genotyping was performed from blood drawn at the in-clinic examination.^11^ Medical conditions were determined for each participant by medical record abstraction using the REP medical records linkage system.^11,12^

### Statistical analysis

Associations between baseline plasma or CSF NfL with demographics (age, sex, education, *APOE*), medical conditions (hypertension, diabetes), or clinical diagnosis (CU vs MCI) were examined using Spearman rank correlation for continuous variables and Kruskal-Wallis tests for dichotomous variables. We used linear mixed regression models with random subject-specific intercepts and slopes for time to examine the cross-sectional and longitudinal associations between baseline plasma or baseline CSF NfL with neuroimaging or cognitive outcomes, adjusting for age, sex, and education. In additional models, we excluded participants with only a 15-month follow-up (n = 17) or excluded MCI cases (n = 15) at baseline. All NfL measures were *z* log-transformed for the linear mixed regression models to normalize the distributions and to utilize the same units for each variable to adequately compare the effect sizes for each outcome. Linear regression models were used to examine change in log-transformed plasma NfL and change in the neuroimaging and cognitive outcomes after adjusting for baseline log-transformed plasma NfL, age, sex, education, and time between visits. All models examining hippocampal volume also adjust for total intracranial volume. Amyloid PET was log-transformed in all analyses. Statistical analyses were completed using SAS, version 9.4 (SAS Institute Inc., Cary, NC), and R, version 3.4.1. A 2-tailed *p* < 0.05 was considered significant.

### Data availability

Data from the MCSA, including data from this study, are available upon request.

## Results

The baseline characteristics of the 79 participants are described in table 1. Participants were a median age of 76 years, 52 (66%) were men, and 15 (19%) had a clinical diagnosis of MCI. At baseline, plasma NfL levels increased with increasing age (Spearman ρ = 0.588, *p* < 0.001) and were higher in those with hypertension compared to without (median 49.2 vs 36.0 pg/mL, *p* = 0.022). However, NfL levels did not differ by sex, education, *APOE* ɛ4 genotype, or diabetes. The 15 MCI participants had higher plasma NfL levels compared to the 64 CU participants (median 50.8 vs 42.2 pg/mL, *p* = 0.181), but the results were not significant. Similarly, baseline CSF NfL levels increased with increasing age (Spearman ρ = 0.363, *p* = 0.001) but levels did not differ by sex, education, MCI diagnosis, *APOE* ɛ4 genotype, hypertension, or diabetes. There was a moderate correlation between plasma and CSF NfL (Spearman ρ = 0.568, *p* < 0.001). The strength of this correlation did not differ by age (<75 vs ≥ 75 years).

**Table 1:**
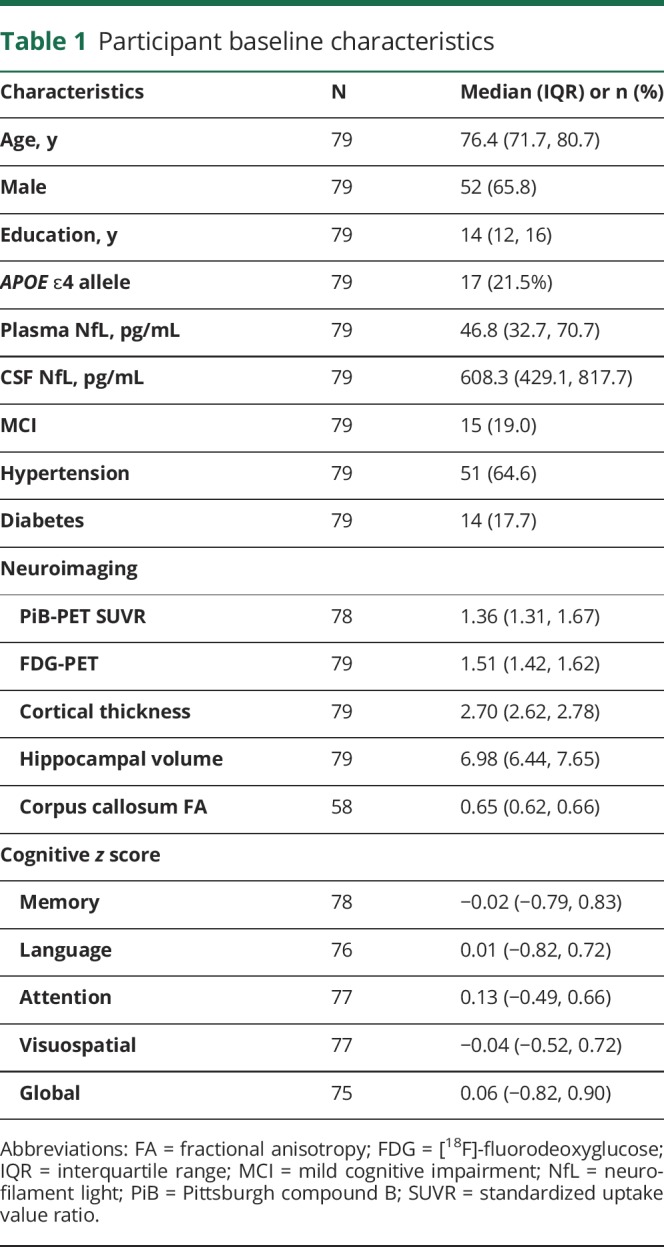
Participant baseline characteristics

The cross-sectional and longitudinal associations between baseline *z* log-transformed plasma or CSF NfL and neuroimaging and cognitive measures are shown in table 2 and the figure, A. In multivariable models adjusting for age, sex, and education, there were no significant cross-sectional associations between baseline plasma (table 2) or baseline CSF (table 3) NfL and baseline neuroimaging or cognitive measures. Longitudinally, higher levels of baseline plasma NfL were significantly associated (*p* < 0.05) with declines in hippocampal volume, cortical thickness, FDG-PET, corpus callosum FA, and global cognitive *z* score. Baseline plasma NfL was not associated with change in amyloid PET. By comparison, although the coefficients for all longitudinal associations between baseline CSF NfL and either neuroimaging or cognitive outcomes were similar and not statistically different, baseline CSF NfL was only significantly associated with declines in cortical thickness and corpus callosum FA. In additional analyses, we restricted the sample to the 64 CU participants at baseline (figure, B) or to the 62 participants with a 30-month follow-up (figure, C) and the results remained the same.

**Table 2:**
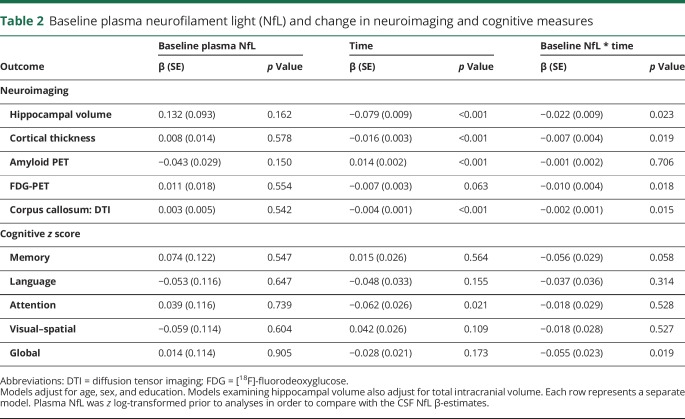
Baseline plasma neurofilament light (NfL) and change in neuroimaging and cognitive measures

**Figure:**
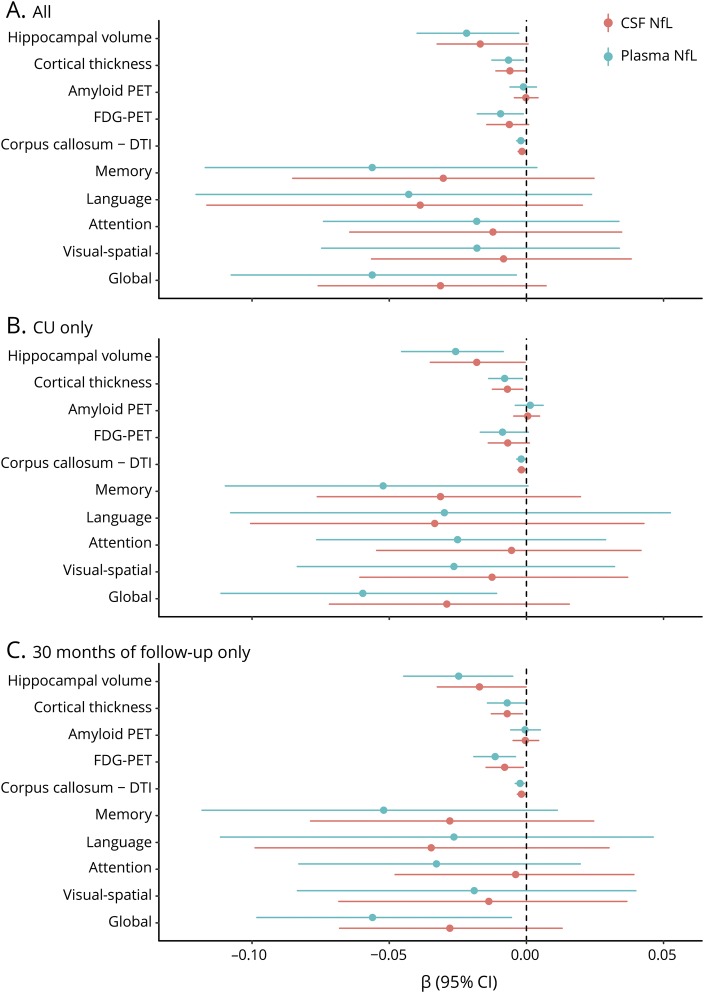
Longitudinal associations between baseline z log-transformed plasma or CSF neurofilament light (NfL) and neuroimaging and cognitive measures (A) Everyone. (B) The 64 cognitively unimpaired (CU) participants at baseline. (C) The 62 participants with a 30-month follow-up. CI = confidence interval; DTI = diffusion tensor imaging; FDG = [^18^F]-fluorodeoxyglucose.

**Table 3:**
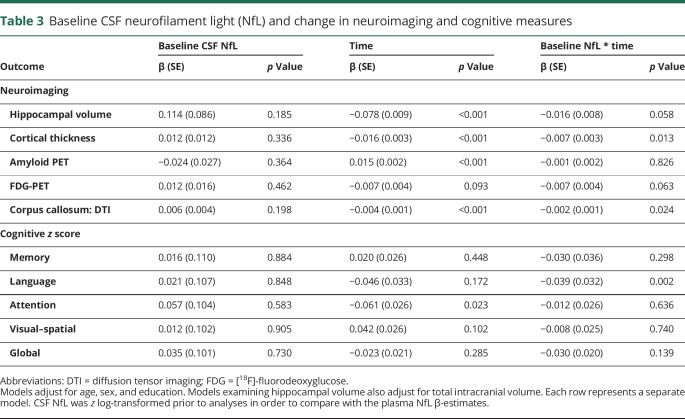
Baseline CSF neurofilament light (NfL) and change in neuroimaging and cognitive measures

All 79 participants had a repeat blood draw and plasma NfL measure 15 or 30 months later, concurrent with their follow-up neuroimaging measure. The median (interquartile range) of plasma NfL increased from 46.8 pg/mL (32.7, 70.7) to 58.8 pg/mL (39.6, 77.4) over the follow-up; the median increase did not differ by baseline diagnosis. Table 4 shows the association between change in log plasma NfL and change in the neuroimaging or cognitive measures after adjustment for baseline log plasma NfL, age, sex, education, and time between visits. Increasing levels of log plasma NfL over the short follow-up were associated with increasing levels of log amyloid PET (b = 0.036, *p* = 0.030) as well as decreasing attention (b = −0.476, *p* = 0.019) and global (b = −0.383, *p* = 0.019) *z* scores.

**Table 4:**
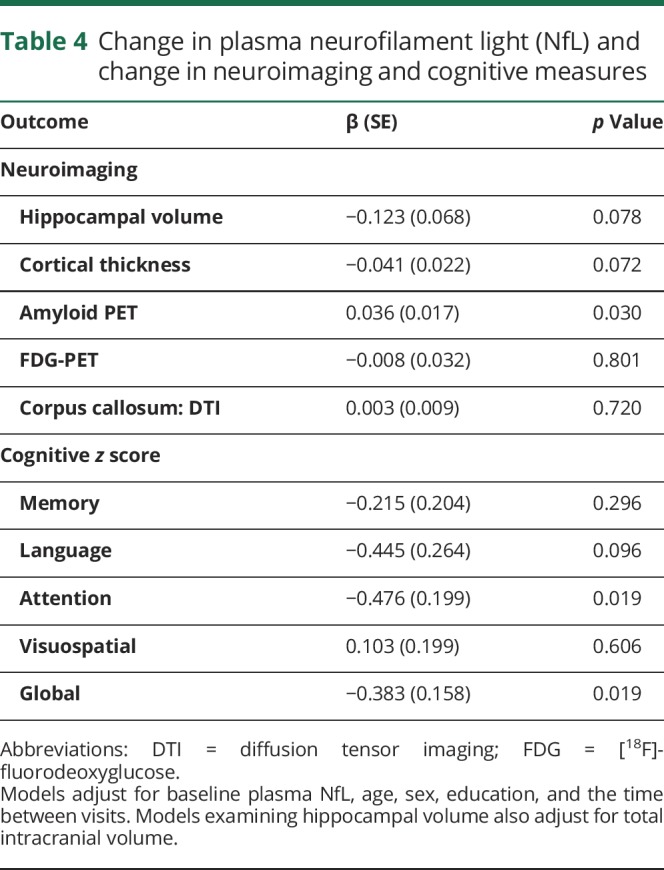
Change in plasma neurofilament light (NfL) and change in neuroimaging and cognitive measures

## Discussion

In the present study, we examined and compared the effect sizes of baseline plasma and baseline CSF NfL levels for short-term (15 to 30 months) change in neuroimaging and cognitive outcomes. We found that baseline plasma and baseline CSF NfL were similarly associated with short-term declines in imaging measures of neurodegeneration and with global cognition, but not with change in amyloid ligand retention on PET. We also found that an increase in plasma NfL levels over the short follow-up was associated with significant declines in both attention and global *z* scores and an increase in amyloid ligand retention on PET. These results suggest that plasma NfL is a useful marker for the short-term prognosis of nonspecific neurodegenerative and cognitive changes.

We did not find a cross-sectional association between either plasma or CSF NfL and any neuroimaging or cognitive measure. Although plasma and CSF NfL levels were higher in the MCI participants compared to CU, they were also not significantly different. In Alzheimer's Dementia Neuroimaging Initiative (ADNI) cross-sectional data with a larger sample size, patients with MCI had significantly higher plasma NfL levels compared to CU. However, notably, there was not a cross-sectional association between plasma NfL and cognition when analyses were restricted to the CU group.^3^ This finding is in line with the current results because the majority of our participants were CU at baseline.

Previous longitudinal studies have shown that either plasma or CSF NfL is a prognostic marker for change in cognition and imaging measures of neurodegeneration or white matter integrity, independent of elevated brain amyloid.^3,4,26^ Our longitudinal results, showing that both plasma NfL and CSF NfL are associated with neurodegeneration and cognitive decline, are consistent with these findings. In addition, our observed correlation between plasma and CSF NfL (Spearman ρ = 0.568) was similar to the correlation found in the ADNI (Spearman ρ = 0.590), but lower than what has been reported in, for example, multiple sclerosis, where there is a greater range of concentrations in relation to disease activity.^8^

Despite the consistently observed prognostic value of baseline plasma or CSF NfL for change in cognitive and imaging measures, few studies have compared their effect sizes. Only one cross-sectional study of ADNI patients reported that plasma and CSF NfL similarly correlated with the same regions of cortical and subcortical thickness, but the effect sizes were not compared.^27^ Because CSF NfL is a more direct measure of subcortical axon degeneration than plasma NfL, we would have hypothesized that CSF NfL is more strongly associated with cognitive and neuroimaging outcomes than plasma NfL. Instead, we found more statistically significant associations for plasma NfL compared to CSF NfL for neuroimaging or cognitive outcomes. However, although there were more statistically significant associations for plasma NfL, the effect sizes were similar to CSF NfL and not statistically different. There are a few explanations as to why we may have obtained these results. First, we used different assays for plasma and CSF because of the difference in concentrations between the 2 mediums. Thus, it is possible that there was more variability or other differences in the CSF assay compared to the plasma assay. Second, our sample size was small, which could have led to false-positive findings with respect to plasma NfL and false-negative findings with respect to CSF NfL. Additional longitudinal studies with concurrent measures of baseline CSF and plasma NfL and larger sample sizes are needed to validate our finding. Finally, it is possible that CSF and plasma NfL may be differentially altered over the course of clinical progression, similar to the observation that CSF amyloid-β42 is thought to change earlier than amyloid PET. Serial assessments of both plasma and CSF NfL are needed to determine whether the markers differentially change in relation to clinical progression.

Cross-sectional studies show that both plasma and CSF NfL increase with age,^3,4,26^ which we also replicated in the current study. Longitudinally, plasma NfL levels intraindividually increased over the 15- to 30-month follow-up. The quantitative increase did not differ by baseline cognitive status (CU vs MCI), but the sample size was small, the time period was short, and there were very few clinical transitions (CU to MCI or MCI to dementia). Notably, increasing levels of plasma NfL were significantly associated with declines in both attention and global cognitive *z* scores, even over the short follow-up. These results suggest that plasma NfL may track with cognitive decline. If this finding is replicated, plasma NfL could be utilized as a clinical trial endpoint or marker of disease progression for AD and other neurodegenerative diseases. Interestingly, we also found that increasing plasma NfL was significantly associated with increasing amyloid PET over the short follow-up. This was a bit surprising given that NfL is thought to be a nonspecific marker of neurodegeneration. Although not statistically significant (*p* = 0.07), increases in plasma NfL were also associated with declines in hippocampal volume and cortical thickness over the short follow-up. Thus, it is possible that the observed relationship with amyloid PET is reflecting ongoing AD-associated neurodegeneration. A larger sample size is needed to better understand the longitudinal relationship between change in plasma NfL and change in AD-associated neuroimaging measures.

Strengths of the study include the community-based population with concurrent plasma and CSF NfL and neuroimaging. There are, however, some limitations. First, although the current study is the largest study to date examining serial plasma and neuroimaging data, the overall sample size was small and larger studies are needed. Second, the time between consecutive visits with imaging and plasma NfL was 15 or 30 months, a period in which little cognitive or brain neuroimaging changes may occur in participants without dementia. Regardless, even though the follow-up was short, we still found that change in plasma NfL was associated with worsening in cognition and amyloid PET.

Our results suggest that elevated plasma NfL is a prognostic marker of cognitive decline and imaging measures of neurodegeneration and has similar effect sizes to CSF NfL. Change in plasma NfL also tracked with short-term cognitive change, and may be a potential marker of disease progression. Plasma NfL is more clinically suitable than CSF NfL, more feasible at the population level for serial measures to assess disease progression, and similarly represents neurodegenerative and cognitive changes. Future studies will need to determine whether plasma NfL tracks with cognitive or imaging changes across the clinical spectrum or is a better measure in either the early or later clinical phases of the disease.

## Glossary

AD: Alzheimer disease
ADNI: Alzheimer's Dementia Neuroimaging Initiative
CU: cognitively unimpaired
DTI: diffusion tensor imaging
FA: fractional anisotropy
FDG: [^18^F]-fluorodeoxyglucose
MCI: mild cognitive impairment
MCSA: Mayo Clinic Study of Aging
NfL: neurofilament light
PiB: Pittsburgh compound B
REP: Rochester Epidemiology Project
SUVR: standardized uptake value ratio
